# Supplementary material for: Wild-type C9orf72 expression is a genetic modifier of C9-ALS survival
Source: medRxiv. 2026 Feb 9:2026.02.06.26345684. Preprint. [Version 1] doi: 10.64898/2026.02.06.26345684 (PMC12919128; doi:10.64898/2026.02.06.26345684)
Supplement: Supplement 2 [file NIHPP2026.02.06.26345684v1-supplement-2.pdf]

medRxiv preprint doi: https://doi.org/10.64898/2026.02.06.26345684; this version posted February 9, 2026. The copyright holder for this preprint (which was not certified by peer review) is the author/funder, who has granted medRxiv a license to display the preprint in perpetuity.
It is made available under a CC-BY 4.0 International license .

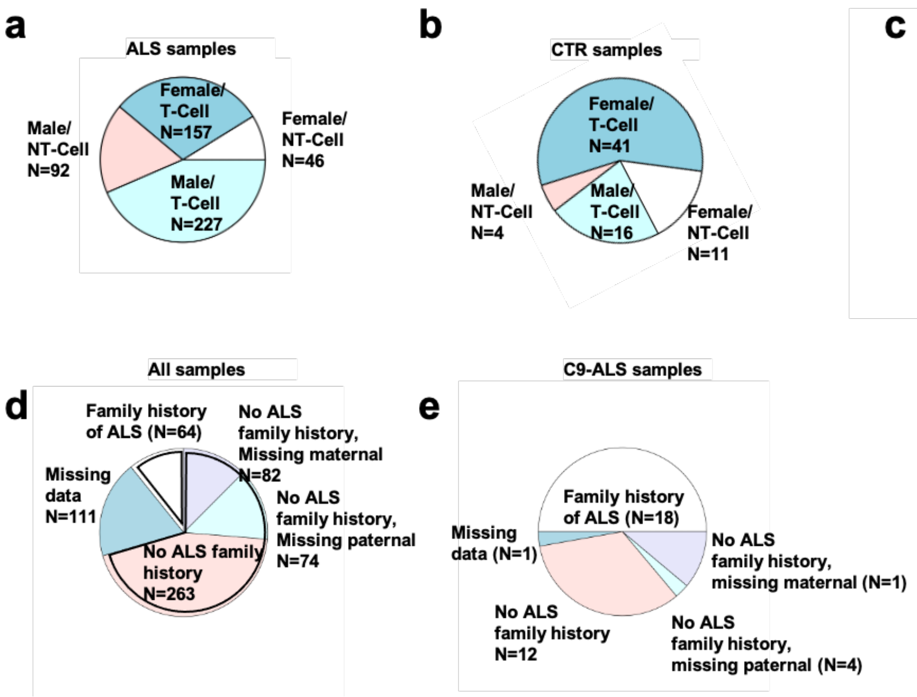


Figure S1. Demographics of AALS subjects used in QTL analysis. A) Pie chart depicting demographics of ALS samples by sex and iPSC cell type of origin (T-cell/NT-cell derived). B) Same as (A), but for healthy control lines. C) Proportions by ancestry. EUR: European ancestry, EAS: East Asian ancestry, AMR: Native American ancestry, AFR: African ancestry, SAS: Southeast Asian ancestry. D) Distribution of data availability on family history for all ALS and control samples. “No ALS family history” means that data for at least one maternal and one paternal relative is present. E) Distribution of family history availability for all subjects carrying the C9orf72 mutant hexanucleotide repeat expansion.

medRxiv preprint doi: https://doi.org/10.64898/2026.02.06.26345684; this version posted February 9, 2026. The copyright holder for this preprint
(which was not certified by peer review) is the author/funder, who has granted medRxiv a license to display the preprint in perpetuity.
It is made available under a CC-BY 4.0 International license .

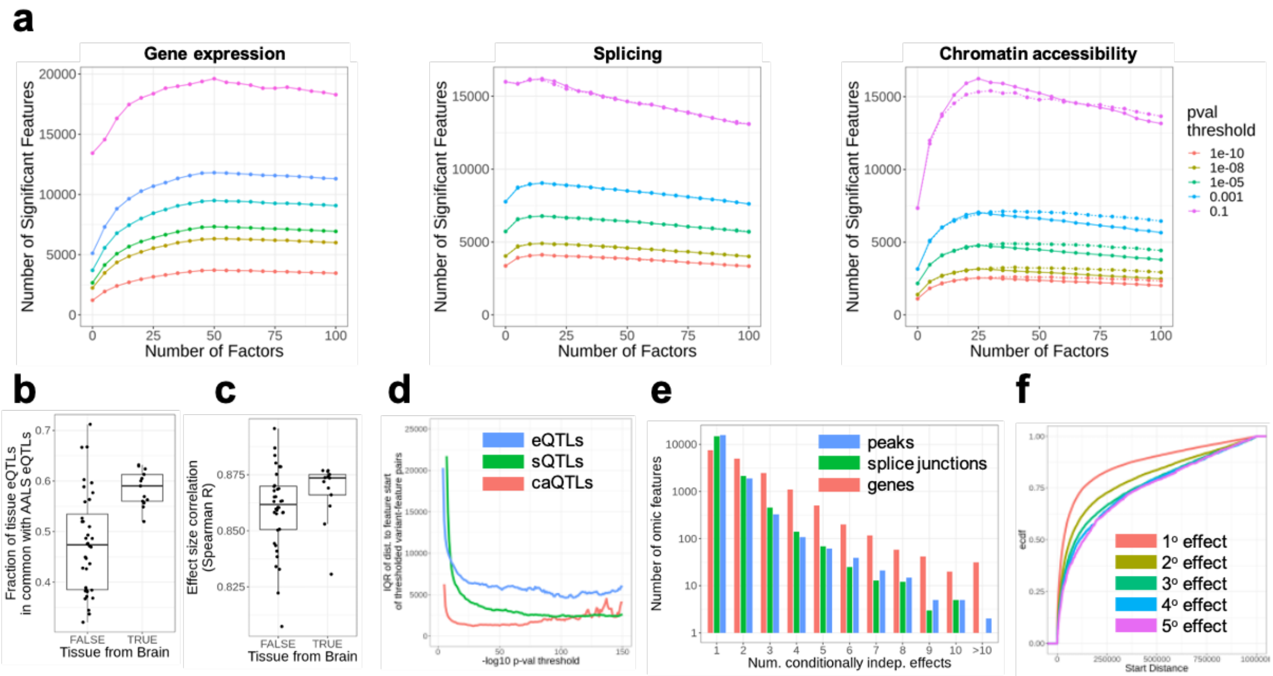


Figure S2. Extended QTL characterization and accounting for batch effects. A) Plots showing the number of significant features (y axis) as a function of the number of top PEER factors regressed out (x axis) for eQTLs (left), sQTLs (middle), and caQTLs (right). Colored lines represent p-value thresholds (see inserted legend). B) Fraction overlap between GTEx Consortium tissue-specific eQTLs and AALS eQTLs, for 47 tissues. C) Effect size correlation between GTEx Consortium tissue-specific eQTL and AALS eQTLs, for 47 tissues. D) Interquartile range of the distance to feature for eQTLs, sQTLs, and caQTLs as a function of p-value threshold. Colored lines represent various QTL (see inserted legend). E) Distribution of the number of conditionally independent effects (genomic variants) influencing gene expression, splicing, and chromatin accessibility. Colored lines represent various effectors (see inserted legend). F) Empirical cumulative distribution function of the distance to the transcription start site for primary eQTLs ($1^{o}$ effect, top driver of variation), secondary eQTLs ($2^{o}$ effect, second driver of variation), etc. (see methods). Colored lines represent various effects (see inserted legend).

medRxiv preprint doi: https://doi.org/10.64898/2026.02.06.26345684; this version posted February 9, 2026. The copyright holder for this preprint
(which was not certified by peer review) is the author/funder, who has granted medRxiv a license to display the preprint in perpetuity.
It is made available under a CC-BY 4.0 International license .

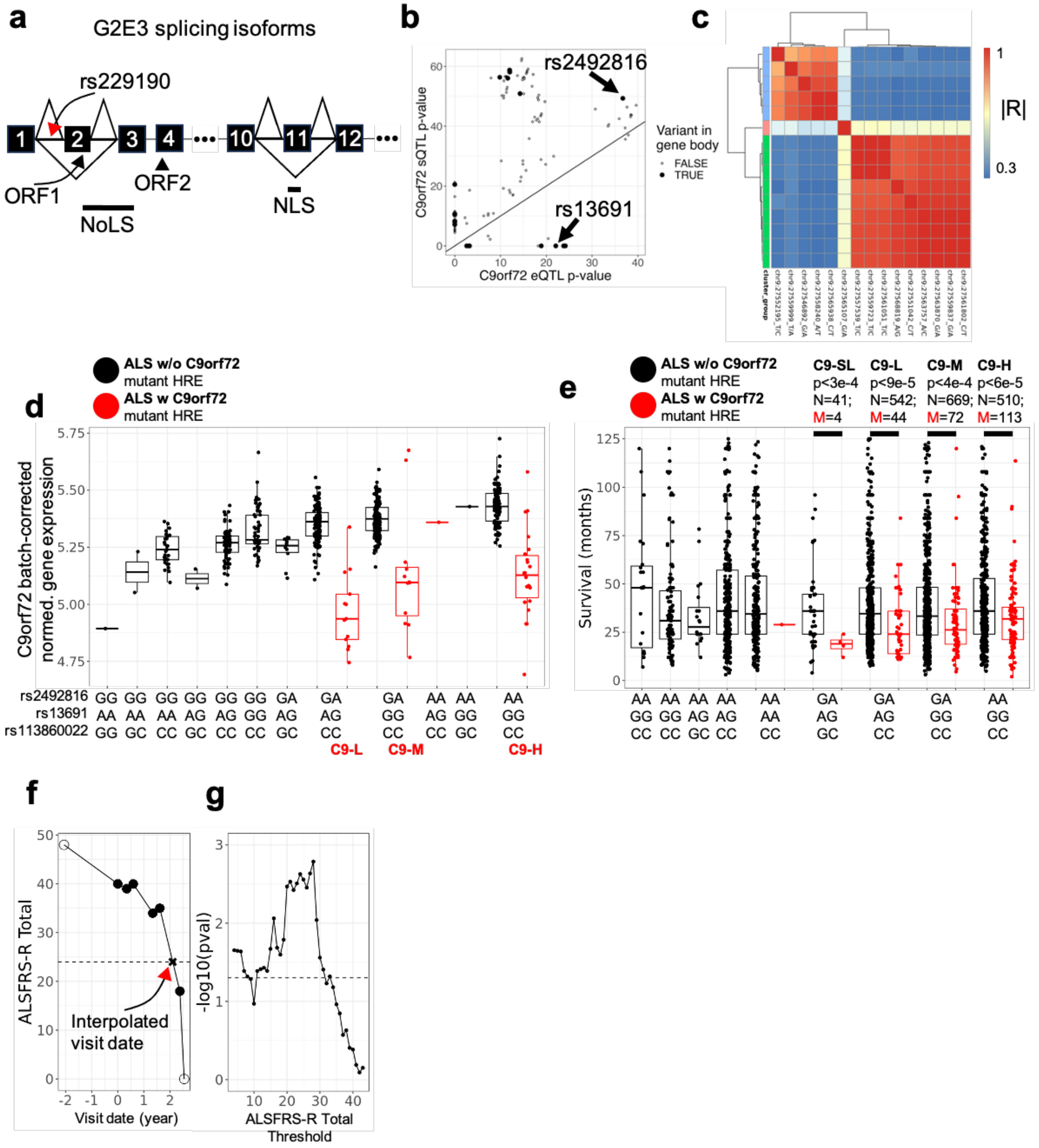


Figure S3: Comparison of significant QTLs and significant ALS GWAS variants. A) Schematic illustrating alternative splicing of *G2E3*. ORF: open reading frame. NoLS: Nucleolar localization sequence. NLS: nuclear localization sequence. Skipping of exon 2 is significantly associated with rs229190 genotype. There is frequent skipping of exon 11, but no significant associations with genotype could be found. B) *C9orf72* sQTL p-values plotted against *C9orf72* eQTL p-values. Large solid dots correspond to QTLs in the pre-mRNA transcript; small unfilled dots are not. C) Correlation matrix of genomic variants from Figure 3f that fell within the gene body and were either highly significant sQTLs or eQTLs. Variants in cluster group 1 were only highly significant sQTLs, variants in cluster group 2 were only highly significant eQTLs, and the variant in cluster group 3 was both a highly significant eQTL and sQTL. D) Batch-corrected normalized gene expression of *C9orf72* plotted as a function of the combined genotypes of rs2492816 (top genotype), rs13691 (middle genotype), and rs113860022 (bottom genotype). Red dots indicate carriers of the mutant repeat expansion;

medRxiv preprint doi: https://doi.org/10.64898/2026.02.06.26345684; this version posted February 9, 2026. The copyright holder for this preprint (which was not certified by peer review) is the author/funder, who has granted medRxiv a license to display the preprint in perpetuity.
It is made available under a CC-BY 4.0 International license .

black dots indicate non-C9 ALS patients and healthy controls. C9-L, C9-M, and C9-H label annotate the C9-ALS patients by the haplotype group of their wild-type allele (see Figure 4E). E) Disease survival plotted as a function of the combined genotypes of rs2492816 (top genotype), rs13691 (middle genotype), and rs113860022 (bottom genotype) for all ALS patients with available data in the ALS Compute database. Genotypes are plotted to match Figure 4E to the extent possible. F) Example estimation of time to ALSFRS-R = 24. Solid points indicate gathered longitudinal data. Empty point indicates date of death, anchored at 0. Dashed horizontal line corresponds to ALSFRS-R=24. G) The p-value as calculated for Figure 4j, but with a range of different ALSFRS-R total thresholds. Dashed line corresponds to p-value 0.05.

medRxiv preprint doi: https://doi.org/10.64898/2026.02.06.26345684; this version posted February 9, 2026. The copyright holder for this preprint (which was not certified by peer review) is the author/funder, who has granted medRxiv a license to display the preprint in perpetuity.
It is made available under a CC-BY 4.0 International license .

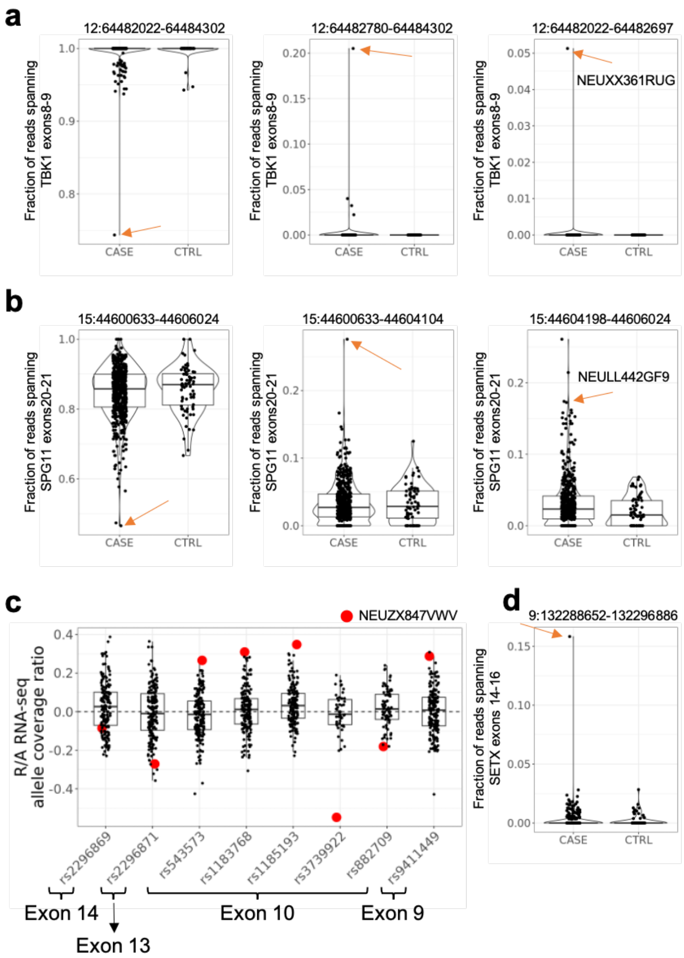


Figure S4. Rare splicing in ALS genes. A) Percent spliced in for cryptic exon in *TBK1* found in sample NEUXX361RUG. Red arrow indicates NEUXX361RUG. B) Percent spliced in for cryptic exon found in *SPG11* in sample NEULL442GF9. Red arrow indicates NEULL442GF9. C) RNA-seq reference/alternate allele imbalance across all samples for eight SNPs in SETX at which NEUZX847VWV (red) is heterozygous. D) Percent spliced in for *SETX* exon 15. Red arrow indicates NEUZX847VWV.
